# Microscale Quantitative Analysis of Polyhydroxybutyrate in Prokaryotes Using IDMS

**DOI:** 10.3390/metabo7020019

**Published:** 2017-05-17

**Authors:** Mariana Itzel Velasco Alvarez, Angela ten Pierick, Patricia T. N. van Dam, Reza Maleki Seifar, Mark C. M. van Loosdrecht, S. Aljoscha Wahl

**Affiliations:** 1 Cell Systems Engineering Group, Department of Biotechnology, Delft University of Technology, 2629 HZ Delft, The Netherlands; M.I.VelascoAlvarez@tudelft.nl (M.I.V.A.); P.T.N.vanDam@tudelft.nl (P.T.N.v.D.); Reza.Maleki-Seifar@dsm.com (R.M.S.); M.C.M.vanLoosdrecht@tudelft.nl (M.C.M.v.L.); 2 BioAnalytical Chemistry, Department of Chemistry and Pharmaceutical Sciences, Vrije Universiteit Amsterdam, 1081 HV Amsterdam, The Netherlands; a.ten.pierick@vu.nl

**Keywords:** polyhydroxybutyrate, 3-hydroxy butyrate, GC-MS, ^13^C-labeled internal standard, isotope dilution, *Escherichia coli*

## Abstract

Poly(3-hydroxybutyrate) (PHB) is an interesting biopolymer for replacing petroleum-based plastics, its biological production is performed in natural and engineered microorganisms. Current metabolic engineering approaches rely on high-throughput strain construction and screening. Analytical procedures have to be compatible with the small scale and speed of these approaches. Here, we present a method based on isotope dilution mass spectrometry (IDMS) and propanolysis extraction of poly(3-hydroxybutyrate) from an *Escherichia coli* strain engineered for PHB production. As internal standard (IS), we applied an uniformly labeled ^13^C-cell suspension, of an *E. coli* PHB producing strain, grown on U-^13^C-glucose as C-source. This internal ^13^C-PHB standard enables to quantify low concentrations of PHB (LOD of 0.01 µg/g_CDW_) from several micrograms of biomass. With this method, a technical reproducibility of about 1.8% relative standard deviation is achieved. Furthermore, the internal standard is robust towards different sample backgrounds and dilutions. The early addition of the internal standard also enables higher reproducibility and increases sensitivity and throughput by simplified sample preparation steps.

## 1. Introduction

Since the discovery of PHB accumulating microorganisms, different approaches have been proposed for a precise and accurate measurement of the PHB content [1,2]. The first method was developed by Lemoigne [3], which was followed by a series of improvements. Especially, different reagents for extraction like dichloroethane, chloroform [4], or free chlorinated solvents like propanol or butanol [5] have been developed. Nowadays, one of the most commonly used methods is propanolysis and GC-based quantification [6,7,8,9]. The method can be applied to a wide range of microorganisms [10,11]. Next to propanolysis an approach based on derivatization with *N*-tert-butyldimethylsilyl-*N*-methyltrifluoroacetamide (MTBSTFA) and GC-MS/MS has been proposed [12]. A comparison of the described methods including required sample amounts and accuracy can be found in Table 1. Unfortunately, not all methods were fully documented with respect to detection limits and accuracies—therefore, partly, the lowest calibration point rather than detection limit is reported.

In this study, the LOD reported is based on a split 1:500 injection meaning that only 2 nL is injected on the column, the GC-MS/MS method is used in SL (splitless) mode, which means that 0.1 µL is injected. Therefore, there is also the possibility of lowering the LOD at least 100 times.

There are two main limitations in the current methods: (1) laborious and time consuming sample preparation and (2) the requirement of several milligrams of biomass [1]. In view of high-throughput experimentation, a fast and small-scale method is needed. Especially, the cultivation volume will be limited and only small sample volumes can be obtained. Further, a high amount of samples is only feasible with a robust and simple protocol [16,17].

Here, we present an approach that is robust towards the cellular matrix and requires small sample volumes, especially the weighing step of samples and standards for the PHB quantification. The major difference to previous methods is the application of a labeled internal standard [18,19].

## 2. Materials and Methods

### 2.1. Strain and Preculture

The *E. coli* MG1655-PHB strain was obtained from a transformation with a plasmid (pBBR1MCS-2-*phaCAB*), constructed in the University of São Paulo (Brazil) containing the genes of *Ralstonia eutropha* for poly(3-hydroxybutyrate) production [20]. The stock cells were grown in mineral medium and aliquots containing glycerol were stored at −80 °C until further use for inoculation (80% *v*/*v* glycerol).

### 2.2. Production of the Internal Standard, ^13^C Labeled Cell Suspension Containing PHB

A shake flask cultivation of 0.1 L using *E. coli* MG1655-PHB was grown at 37 °C and 220 rpm. A low ammonium minimal medium concentration composition for one liter was used: 2.0 g (NH_4_)_2_SO_4_, 2.0 g KH_2_PO_4_, 0.5 g MgSO_4_·7H_2_O, 0.5 g NaCl, 0.001 g vitamin (Thiamine-HCl), 0.05 g kanamycin and 1 mL of trace elements solution (Sctra-1) [21]. The carbon source was U-^13^C-glucose 99% purchased from Cortecnet (Paris, France) in a concentration of 20 g/L. The pH of the medium was adjusted to 7 with 1 M K_2_HPO_4_, and 8.37 mg/mL of MOPS ((3-(*N*-morpholino)propanesulfonic acid) was added as a buffer. After adjusting the pH, the medium was sterilized using a 0.2 µm pore size filter, cellulose acetate (FP 30/0.2, Whatman GmbH, Dassel, Germany). The cultivation was stopped after four days (glucose was fully consumed). The broth was centrifuged and resuspended in milli-Q water (EMD Millipore HQ, Billerica, MA, USA). The cell suspension was then distributed into several falcon tubes and kept at −80 °C until further usage. For simplicity, we will call the labeled cell suspension (containing PHB) IS-^13^C-PHB.

### 2.3. Calibration Lines of 3-HB

Sodium 3-hydroxybutyrate (95.2% purity) was purchased from Sigma Aldrich (Darmstadt, Germany). The concentration range of the calibration lines were from 0.001 to 11 mmol/L. The (PHB)_1_ concentration of the labeled cell suspension (IS-^13^C-PHB) was determined from the calibration lines (and respective standard volumes). A concentration of 9.3 mmol/L was obtained.

A duplicate of calibration lines was performed to check reproducibility within the same concentration range. The standards were measured in triplicate by injection from the same vial.

In addition, a calibration without the addition of IS-^13^C-PHB was performed (in duplicate).

### 2.4. Cell Dry Weight Measurements

The cell dry weight (CDW) in g/L was determined in triplicate for each shake flask. The samples, 5 mL of cell broth, were filtrated through Pall membrane filters purchased from Sigma Aldrich (Darmstadt, Germany), of a pore size of 0.2 µm. The cell broth was pipetted, on the pre-weighted membranes. After 48 h at 60 °C of drying, the membranes were weighted. The difference in weight divided by the amount of sample taken (5 mL), is defined as the cell dry weight.

### 2.5. Quantification of PHB

One milliliter of broth was transferred into a 1.5 mL microcentrifuge Eppendorf tube and centrifuged. The pellet was resuspended in a mixture of 200 µL of the IS-^13^C-PHB and 500 µL milli-Q water and transferred to a silanized glass GC vial with screw cap (w/spot 2 mL, Agilent Technologies, Santa Clara, CA, USA). The sample was then freeze-dried overnight.

**Figure 1:**
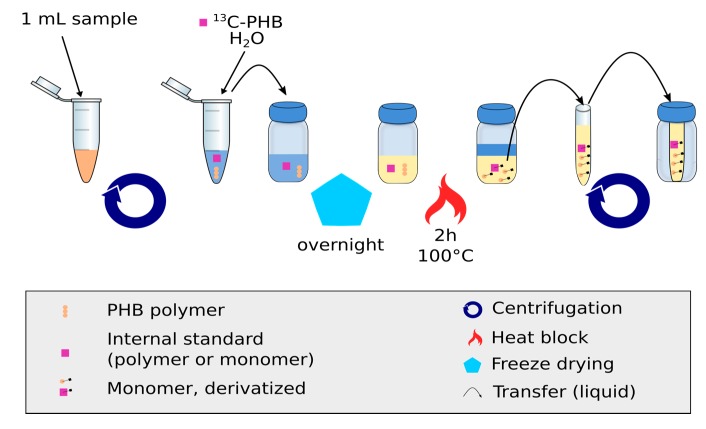
Sample processing steps for the measurement of (PHB)_1_ using the IDMS approach. Broth is centrifuged and re-suspended to remove extracellular matrix. After freeze-drying, the sample is further cleaned and derivatized.

The dried samples were derivatized using the propanolysis reaction [6]. i.e., 50 μL of 2 g/L of benzoic acid (Acros Organics, Antwerp, Belgium) in 1-propanol (99.9% Thermo Fisher Scientific, Waltham, MA, USA), 100 µL of 3:7 HCl:1-propanol, and 150 µL of 1,2-dichloroethane (99.9% Thermo Fisher Scientific, Waltham, MA, USA). The screw cap (w/spot 2 mL, Agilent Technologies, Santa Clara, CA, USA) vials were then placed in a heating block at 100 °C for 2 h. During the heating, the vials were shortly vortexed every 30 min. After cooling down, the lower organic phase was transferred into a 1 mL shell vial (8 mm × 40 mm; Grace Drive, Columbia, MD, USA) and 150 µL of 1,2-dichloroethane and 300 µL of milli-Q water were added. The mixture was vortexed for 5–10 s and centrifuged for 1 min at 10,000× *g*. The lower organic phase was recovered in a fresh shell glass vial. Remaining traces of water were removed by the addition of about 5 mg of Na_2_SO_4_ and centrifuged for 1 min at 10,000× *g*. A volume of 120 µL was transferred into the GC vial with an insert (250 μL pulled point glass Agilent Technologies, Santa Clara, CA, USA) (see Figure 1).

### 2.6. GC-IDMS Condition Analysis

GC–MS measurements were carried out on a 7890A GC coupled to a 5975C Quadrupole MSD (both from Agilent, Santa Clara, CA, USA). The conditions were optimized for the measurement of the metabolites of interest and were set to: 1 μL of derivatized sample was injected with a split ratio of 1:500. Straight ultra-inert glass liners with glass wool were used (Agilent). The Multimode Inlet (MMI; Agilent) temperature was set to 80 °C with a hold time of 3 s. The MMI temperature was raised to 220 °C with a gradient of 720 °C/min and held for 5 min. Subsequently, the temperature was further raised to 300 °C for cleaning purposes.

The column was a Zebron ZB-50 column (30 m × 250 μm internal diameter, 0.25 μm film thickness; Phenomenex, Torrance, CA, USA). The carrier (helium) flow during the analysis was set at 1 mL/min. The GC oven temperature was held for 0.5 min at 80 °C, then raised with 10 °C/min up to 175 °C. At the end of a GC run, the column was back flushed with five column volumes at 300 °C.

The temperature of the transfer line to the MS was set to 250 °C, the MS source to 230 °C and the quadruple to 150 °C. Electron impact ionization (EI) was operated at 70 eV. For quantitative measurements, the MS was used in SIM (Selective Ion Monitoring) mode. The mass resolution was set to 0.6 mass units. To ensure reliable (automatic) peak integration, the dwell times were set to acquire at least 20 points per peak. Raw MS data were processed and integrated using Mass Hunter Quantitative Analysis software for MS (version B.07.00; Agilent). Peak integration was performed with the Agile 2 integrator implemented in Mass Hunter, no base line correction was required. The ^13^C contribution of the ^12^C sample peak was calculated from the natural isotope abundances (0.044%) and taken into account in the concentration calculation [19]. The ^12^C content of the internal standard is included in the offset of the calibration line, and is thus subtracted automatically.

## 3. Results and Discussion

The development of the novel PHB measurement method required a series of experiments to verify the potential use of the proposed IS and the reduction of steps involved in the analytical protocol. Besides establishing calibration lines and the study of a matrix effect, other experiments were performed such as complete assessment of the derivatization time, homogeneity, and simultaneous biomass quantification, which can be found in the Supplementary Materials.

### 3.1. PHB Determination from Small Sample Volume

To test if the extraction and derivatization from small sample volumes would bias the results, the results were compared to the GC-FID based measurement [4]. The results showed a PHB content of 14.4 ± 1.6% with the new method and 12.7 ± 2.0% with the conventional method. The *t*-test (*p* < 0.05) demonstrate no significant difference between methods.

### 3.2. Calibration with Labeled PHB as Internal Standard

Equal amounts of IS-^13^C-PHB were added to each standard of 3-HB. The peak area ratio was then measured by GC-IDMS. Linear calibration lines were obtained from the ratio between the ^12^C-3HB and the IS-^13^C-PHB suspension). The standard deviation of the ratio (^12^C-PHB/IS-^13^C-PHB) measurements was used to calculate the relative and the absolute error: 0.0053 mmol/L and 3 × 10^−5^ respectively. The resulting heteroscedastic error model was used for weighted linear regression.

**Figure 2:**
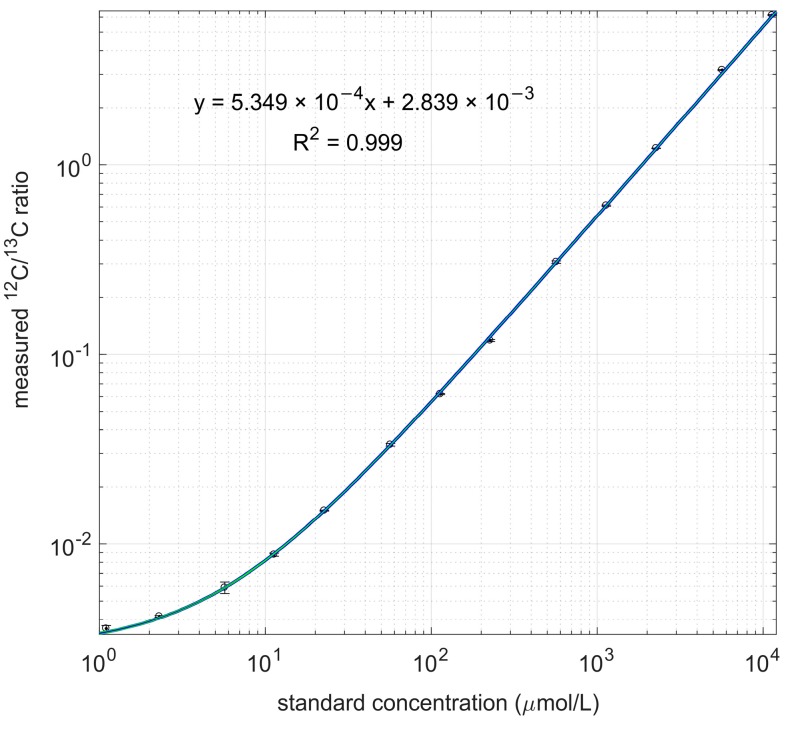
Measured ^12^C/^13^C ratio and linear regression line. Both standard concentration and measured ratio are in log scale.

A calibration line, valid over four orders of magnitude was obtained (Figure 2).

## 4. Validation Experiments

Representative samples were produced by cultivation of *E. coli* MG1655-PHB in minimal medium in aerobic shake flasks. Samples were taken at the end of the cultivation, containing about 1.2 g_DW_/L biomass with a PHB content of about 20% (g/g_DW_). The following analytical tests were performed:

1. Technical reproducibility
2. Sample background impact (matrix effect)
3. Putative influence of the cell matrix

### 4.1. Technical Reproducibility Including Sample Processing

The technical variability of sample processing and measurement was determined by three replicate broth samples from two different cultures (biological duplicate). Each sample was measured three times (analytical variability, see data in the Supplementary Materials, Table S3). The relative standard deviation of the analytical replica measurement was 1.5% while the technical reproducibility (measurement and processing) was around 1.9%.

### 4.2. Putative Influence of Sample Background (Matrix Effect)

In contrast to pure standards, samples may contain interfering compounds. To exclude an influence from the cellular matrix, experiments with diluted matrix and standard addition were performed. Six samples (three replicates from two different cultures) were spiked with a known (PHB)_1_ concentration of 2.3 mmol/L. The recovery of the standard addition was analyzed through comparing the ratios at the standard made with such concentration and the recovered ratios. The recovery obtained was 100 ± 2.2% for the first culture and 103 ± 1.8% for the second culture. To confirm that there is no matrix effect, a one sample *t*-test was performed. The recoveries obtained were compared based on a mean (µ) of 100% recovery. Two hypothesis were formulated: no significant difference was observed in all the recoveries obtained (H_0_) and a difference between the true mean and the values obtained was observed (H_1_). The null hypothesis was accepted (*t*-test, *p* < 0.05), thus we can confirm that there is no effect from the cellular matrix.

### 4.3. Putative Influence of the Cell Matrix

**Figure 3:**
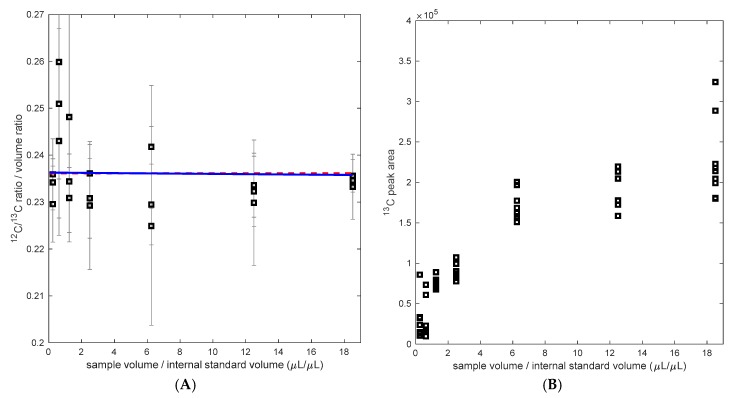
(**A**) Calculated ^12^C/^13^C ratio (measured ratio divided by the volume ratio) as a function of the volume ratio ^12^C/^13^C, in blue the regression assuming an influence of the matrix (H_1_), H_0_ in red. (**B**) ^13^C peak area with different matrix background (sample/internal standard volume).

The impact of cellular matrix was evaluated by preparing different ratios of ^12^C over ^13^C sample volume. The dilution ranged from 0.05 to 3.7 compared to the regular sample preparation (1 mL sample, 200 µL IS-^13^C-PHB). Each sample was prepared in triplicate and measured three times. If there was no matrix effect, the obtained change in ratio is equal to the applied dilution, the concentration of the original sample is independent of the ^12^C to ^13^C volume ratio (Figure 3), and no relation between dilution and the obtained (recalculated) sample concentration (H_0_) is obtained. In case of a matrix effect, a (linear) trend should become visible (H_1_). H_1_ is rejected in a chi-test with *p* > 0.05 [16].

While the ^12^C/^13^C ratio measurement was not influenced by the matrix composition, the measured peak area was influenced. IS-^13^C-PHB led to significantly different signal responses (Figure 3B). As the concentration of cell matrix background and/or the analyte (PHB)_1_ increases, the response of the peak area of ^13^C also increases. Such behavior of either enhancement or suppression has already been reported in other studies using MS (/MS) [22]. The relevance of such matrix effect is reflected on the signal intensity, which can vary from measurement to measurement. For instance, in the sample background analysis (Test 3), it was observed that the peak areas increased by 40% in the standards when the IS-^13^C-PHB was added, compared to samples with no addition of the IS. Thus, the importance of an internal standard is confirmed since the ratio gives more stability to fluctuations in the sample preparation and process. Here, a good balance between saving internal standard and high accuracy in the ratio measurement was found at a volume ratio of 0.2 (sample vs. IS). Clearly, this ratio may vary depending on the concentration determined of the IS-^13^C-PHB but also the sample concentration (biomass concentration and PHB content).

## 5. Conclusions

Using a sensitive detection system (GC-MS) together with an appropriate internal standard, a robust, easy to perform PHB quantification method was achieved. The IS-^13^C-PHB suspension not only corrects for matrix effects, but also variations in the sample preparation process that is suitable for low sample amount measurements. A low sample volume can provide flexibility and convenience for the researcher to design experiments. For instance, low volume cultivation experiments using shake flasks, falcon tubes, or even a microtiter plate.

The technical reproducibility for sample measurements was 1.9%. Including the sample processing steps, a reproducibility of 2% was observed, supporting that the internal standard can correct for sample processing variability [23]. While the reported method was focused on PHB, different polymers are produced by microorganisms. The presented approach can be extended to the quantification of PHV, P4HB (poly(4-hydroxybutyrate)), and other polyhydroxyalkanoates.

## Figures and Tables

**Table 1:** Comparison of most common approaches for PHB quantification in biomass. The concentration is given as molar monomer units (PHB)_1_.

	Method					
	GC-FID [6,12]	GC-MS/MS [12]	HPLC [13]	IC [14]	GC-IDMS [This Study]	Raman Spectroscopy [15]
**Sampled biomass amount (mg_DW_)**	50	50	1000	10	1	1
**Lowest (PHB)_1_ concentration (nmol/L)**	1.16	0.0012	140 ^(a)^	10^6 (a)^	0.011	1395 ^(a)^
**Injection volume**	0.1 µL	0.1 µL	10 µL	−	1 µL	NA
**Used split**	1:100	SL ^(b)^	−	−	1:500	NA
**Internal standard**	Benzoic acid	β-hydroxyoctanoic acid	Adipic acid	NA	U ^13^C-PHB	DNA/Amide I
**External standard**	PHB	3-HBNa ^(c)^	Crotonic acid	3.3:1 PHB:PHV ^(d)^	3-HBNa	PHB

^(a)^ Lowest calibration point; ^(b)^ SL:splitless; ^(c)^ 3-HBNa: sodium 3-hydroxybutyrate; ^(d)^ PHV: Polyhdroxyvalerate.

## Supplementary Materials

The following are available online at www.mdpi.com/2218-1989/7/2/19/s1, Figure S1: Chemical structure of derivatized 3-HB and observed fragments, Figure S2: Observed standard deviation as a function of the measured average ratio in log scale. Based on the observed linearity, a heteroscedastic error model was assumed, Figure S3: Measured ^12^C/BA ratio and linear regression line. Both standard concentration and measured ratio are in log scale, Figure S4: Measured ^12^C/PAA ratio and linear regression line. Both standard concentration and measured ratio are in log scale, Table S1: Preparation of a standard calibration (external) using the following standard concentrations, Table S2: Preparation of a standard calibration (external) using the following standard concentrations, Table S3: Measurement of the (PHB)_1_ concentration from the two different cultivations, each sample was measured in triplicate, Table S4: Comparison of concentration of (PHB)_1_ in different derivatization times, Table S5: Comparison of concentration (PHB)_1_ obtained with and without vortexing, Table S6: Comparison of biomass determination in g/L through freeze drying and filtration.
