# Supplementary material for: Microscale Quantitative Analysis of Polyhydroxybutyrate in Prokaryotes Using IDMS
Source: Metabolites. 2017 May 17;7(2):19. doi: 10.3390/metabo7020019 (PMC5487990; doi:10.3390/metabo7020019)
Supplement: Supplementary file 1 [file metabolites-07-00019-s001.pdf]

Supplementary Materials: Microscale Quantitative Analysis of Polyhydroxybutyrate in Prokaryotes Using IDMS

Mariana Itzel Velasco Alvarez, Angela ten Pierick, Patricia T. N. van Dam, Reza Maleki Seifar, Mark C. M. van Loosdrecht and S. Aljoscha Wahl

1. Fragmentation of Derivatized 3-HB

Two major fragments were identified by GC-MS for PHB analyses: m/z 131 and m/z 145. The fragment m/z 131 was used for the quantification, the mass of the internal standard was m/z 134. The retention time of the fragments was 4.53 minutes.

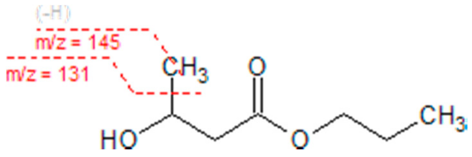

Figure S1. Chemical structure of derivatized 3-HB and observed fragments.

2. Calibration Standards

Table S1. Preparation of a standard calibration (external) using the following standard concentrations (13 points).

	S1	S2	S3	S4	S5	S6	S7
3-HB (mmol/L)	0.001	0.002	0.006	0.011	0.023	0.057	0.113
	**S8**	**S9**	**S10**	**S11**	**S12**	**S13**	
3-HB (mmol/L)	0.227	0.567	1.134	2.268	5.671	11.341	

Table S2. Preparation of a standard calibration (external) using the following standard concentrations (18 points).

	S1	S2	S3	S4	S5	S6	S7	S8	S9
3-HB(mmol/L)	0.001	0.002	0.006	0.011	0.022	0.056	0.111	0.222	0.389
	**S10**	**S11**	**S12**	**S13**	**S14**	**S15**	**S16**	**S17**	**S18**
3-HB(mmol/L)	0.555	0.833	1.110	1.666	2.221	2.776	5.552	8.328	11.104

3. Estimation of the Analytical Standard Deviation of the Measurements ($^{12}C/^{13}C$-PHB)

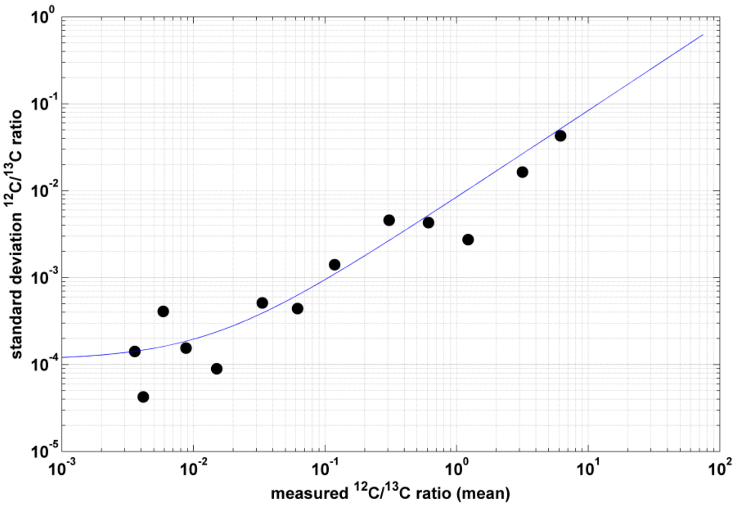

Figure S2. Observed standard deviation as a function of the measured average ratio in log scale. Based on the observed linearity, a heteroscedastic error model was assumed (blue line).

4. Technical and Sample Processing Reproducibility

Table S3. Measurement of the $(PHB)_1$ concentration from the two different cultivations, each sample was measured in triplicate.

	Concentration* (mmol/L)	SE** (mmol/L)	SE (%)	***SE Concentration (mmol/L)	SE %
1	2.754	0.008	0.279		
	2.802	0.042	1.493		
	2.881	0.072	2.503		
Average	2.812	0.041	1.441	0.052	1.86
2	3.054	0.061	2.011		
	3.033	0.057	1.894		
	3.029	0.026	0.848		
Average	3.038	0.048	1.584	0.011	0.37

*Average concentration of three times injection. **Standard deviation of three times injection (analytical reproducibility). ***Standard deviation of the three samples concentration (first column, process reproducibility).

5. Additional Tests on Sample Processing Steps

5.1. Complete degradation of the polymer

The standard method suggests a time for derivatization of two hours. Thus, was desired to confirm whether a complete degradation of the polymer could be achieved in less time. The $(PHB)_1$ concentration at different time points 30, 60, 120, 150, and 180 minutes was tested. One milliliter samples were taken from one shake flask in triplicate, the peak areas obtained were corrected with the IS-^{13}C-PHB. The concentration in the broth was 2.925 ± 0.044. The *t*-test ($p < 0.05$) indicated no significant differences between the time points measured and the concentration in the broth.

Table S4. Comparison of concentration of $(PHB)_1$ in different derivatization times.

Derivatization time (h)	Average (mmol/L)	SE (mmol/L)
0.5	2.685	0.022
1	2.725	0.051
2	2.885	0.046
2.5	2.788	0.037
3	2.826	0.056

5.2. Homogeneity

During derivatization, vortexing is an important step for the complete mixing of the sample. The standard method recommends to vortex every 30 minutes during derivatization. Hence, was determined if the mixing is enough with the boiling itself. Three samples from one shake flask were measured without vortexing during derivatization. The concentrations were corrected with the IS-^{13}C-PHB. The results show that there is no need for vortexing during derivatization. However, this was only tested with *E. coli*, is possible that other species might not deliver the same result if not vortexed.

Table S5. Comparison of concentration $(PHB)_1$ obtained with and without vortexing.

Repeat #	Homogeneity		Reproducibility	
	**Average** (mmol/L)	**SE** (mmol/L)	**Average** (mmol/L)	**SE** (mmol/L)
1	2.793	0.011	2.754	0.008
2	2.820	0.008	2.802	0.042
3	2.853	0.037	2.881	0.072

5.3. Biomass quantification:

In order to know the content of PHB in biomass. Cell dry weight was compared by two methods: filtration and freeze drying.

To determine biomass content by filtration, a 5 mL sample in triplicate was taken from one shake flask. Through freeze drying, first was determined the weight of IS-^{13}C-PHB and PAA only, as a blank. Then the biomass was measured subtracting such value to obtain the actual value of cell dry weight per one milliliter. Each vial was weighted before adding sample and after freeze drying.

There was no significant difference between the two methods used for the biomass determination.

Table S6. Comparison of biomass determination in g/L through freeze drying and filtration.

	Biomass	
	**Freeze drying**	**Filtration**
Average (g_{DW}/L)	1.185	1.300
SE (g_{DW}/L)	0.035	0.0013

6. Calibration Lines with the Internal Standards Benzoic Acid (BA) and Phenyl Acetic Acid (PAA)

6.1. Calibration using Benzoic Acid as Internal Standard

The stock solutions of the internal standards PAA and BA were prepared in a concentration of 0.02 mmol/mL. A volume of 50 µL of each internal standard was added to the samples. The step in which the internal standards were added in the samples differs, BA was added after freeze drying and PAA before freeze drying. The calibration lines obtained were analyzed with and without the addition ofIS-^{13}C-PHB. The internal standard BA was added to compare with the IS proposed in this study. The relative and the absolute error of the data obtained with BA, was calculated with the presence of IS-^{13}C-PHB that was 0.0045 and 0.0004, respectively.

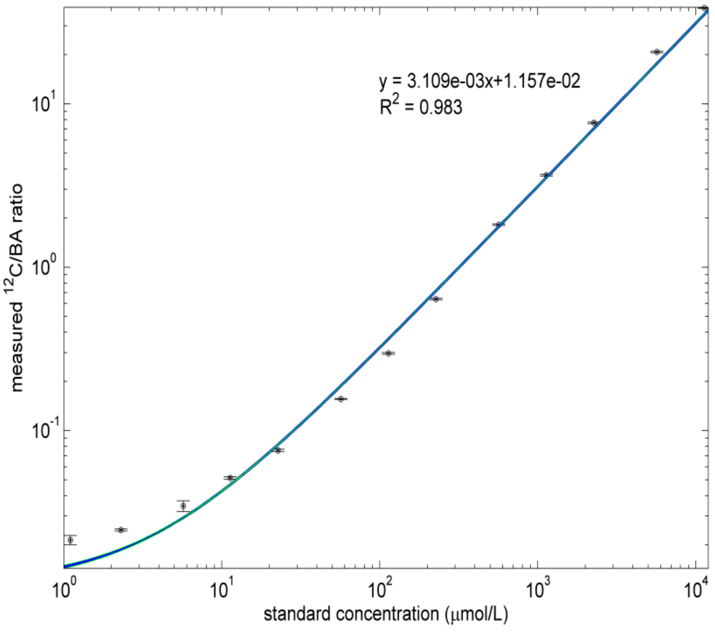

Figure S3. Measured ^{12}C/BA ratio and linear regression line. Both standard concentration and measured ratio are in log scale.

6.2. Calibration using Phenyl Acetic Acid as Internal Standard

The calibration lines obtained were analyzed in the same way as with BA with and without the presence of the IS-^{13}C-PHB. The purpose of adding the internal standard PAA was to have a similar

correction as the ^{13}C-PHB, because was added before freeze drying. The relative and the absolute error with the presence of IS-^{13}C-PHB was calculated that was 0.0137 and 0.00005, respectively. Therefore, such internal standards were not reliable for quantification with GC-IDMS.

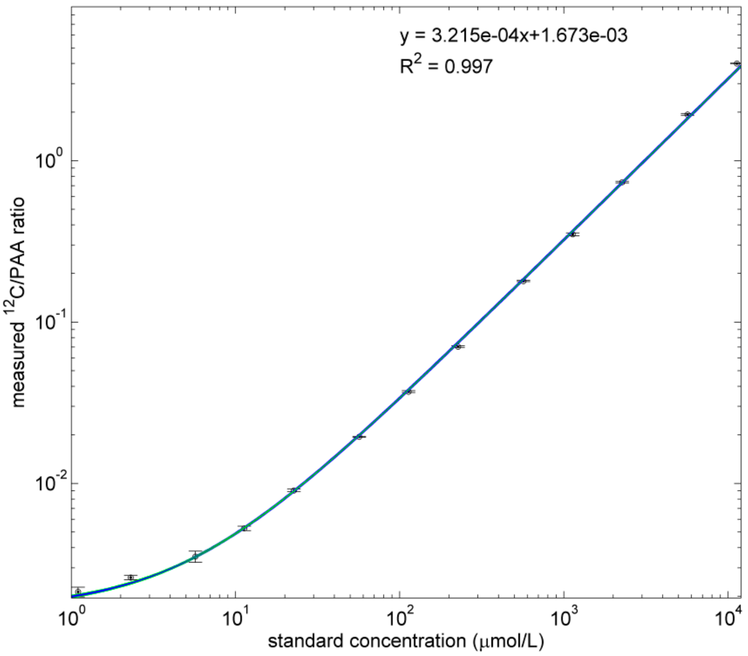

Figure S4. Measured ^{12}C/PAA ratio and linear regression line. Both standard concentration and measured ratio are in log scale.

The internal standards PAA and BA showed lower reproducibility and when spiked with a known amount of $(PHB)_1$ the recoveries were overestimated with BA and underestimated with PAA. The overestimation using BA comes from adding the internal standard after the freeze drying. Therefore, the correction does not take into account losses of the first steps.
